# Richmond Academy of Medicine and Surgery

**Published:** 1895-08

**Authors:** Mark W. Peyser

**Affiliations:** Secretary and Reporter


### Society Reports.

### RICHMOND ACADEMY OF MEDICINE AND SURGERY.

### Regular Meeting.

Dr. Wm. S. Gordon, President, in the Chair.

Dr. Jas. W. Henson read a paper on the "History of Cholelithiasis, and the Anatomy Relating to that Disease." Among other things, he drew attention to the size of the cystic, hepatic and common bile ducts in their various parts, to the size of the opening on the papilla and to the relation of the duct, artery and vein in the transverse fissure of the liver. He stated that there were few muscular fibres in the coat of the gall bladder, and these did not have much power.

Discussion.

Dr. H. H. Levy said from all he could gather, he was very much under the impression that the muscular elements of the gall bladder and ducts were very important, and if so, they must exist to some extent.

Dr. Hugh M. Taylor: In connection with the anatomy of the gall bladder, there are two very important facts of which no mention has been made: 1. The rich system of lymphatics which, in certain conditions, will aid the diffusion of septic matter. 2. The rugæ of the mucosa, which predisposes to inspissation of the bile and formation of stone.

Dr. H. H. Levy read a paper on the "Physiology of the Bile and Gall Bladder and the Etiology of Cholelithiasis." The color of the bile varies, as does its consistency. When fresh, it is slightly viscid, due to the mucus of the bladder and ducts. Normally, it is of a neutral reaction, but may be alkaline, or even acid. The important constituents are: 1. Mucus, prone to decompose and cause alterations in the chemical constitution of the bile. 2. Bile salts, taurocholate and glycocholate of sodium. 3. Pigments, the chief being bilirubin and biliverdin, the former often occurring in combination with alkalies. It is similar to hæmatoidin. 4. Cholesteria in small amounts, characterized by being lævorotatory and occurring in rhombic plates that seem to have one corner broken off. 5. Diastasic ferment. 6. Traces of urea. 7. Inorganic constituents: salts as found in most other secretions, and a considerable amount of $CO_2$ in fresh bile either free, or in combination.

The small amount of cholesterin is noteworthy.

The secretion is not a mere filtration, but a product of the liver cells, always going on, greater at times than at others. It does not pass immediately into the intestine when digestion is not taking place, but regurgitates to the gall bladder, where it is kept until needed.

The quantity secreted in twenty-four hours is about $\frac{1}{14000}$ of the body weight, and the period of greatest flow into the intestine is about three or four hours after the ingestion of food. Circumstances influencing secretion are: 1. Food. Nitrogenous increases it more than vegetable, while fatty foods have no effect. 2. Water in large amounts increases the quantity, but lowers the specific gravity. 3. Other things equal, an increased blood supply increases the quantity, and *vice versa*. 4. Any condition increasing disintegration of the red corpuscles increases the quantity of bile.

The flow is influenced by: 1. The *vis a tergo*. 2. Descent of the diaphragm pressing on the liver in inspiration. Negative pressure produced by inspiration also aids it. 3. Contraction of the muscular fibres of the bladder and ducts. 4. Stimulation of the cord as by passage of food into the stomach and duodenum. In connection, a practical point to note is that a small amount of resistance to overflow is sufficient to cause stagnation of the bile.

DISPOSAL OF THE BILE.—The water aids the maintenance of the softness of the fæces; mucus passes out unchanged; the pigments do not appear in their own forms in the excretions, but as hydrobilirubin, urobilin and stercobilin. The meconiums of the fœtus contain the pigments unchanged. The bile salts are mainly reabsorbed. Cholesterin and lecithin are found in the fæces.

COMPOSITION OF GALL STONES.—Cholesterin constitutes by far the greatest number, but all do not contain it, and often it is mixed with fatty or saponaceous matters, or pigments. Some are composed of bilirubin (mostly in combination with calcium), in strata or masses of cholesterin. Hydrobilirubin may alone form them, as also may glycocholate and taurocholate of calcium. Many are made up of fatty acids and soaps. Mucus and epithelium occasionally constitute small stones, or the nuclei of larger ones. Sometimes they are formed of the oxides of the heavy metals, with occasionally nuclei of globules of mercury, and sometimes there is a chalkstone of the earthy carbonates. The nucleus is mostly composed of a little mucus from the gall bladder. The physical characters are varied, some being white, simple and homogeneous; but more commonly they are mixed either in radiations from the nucleus, or in concentric rings around it. The crust is nearly always of pure cholesterin, but sometimes it is formed of fatty acids, coloring matter and cholesterin.

ETIOLOGY.—The oldest supposable cause for the formation of gallstone is inspissation; but it is rare to find all the constituents of the bile, except water, in a single stone. Other theories are, lessened secretion of sodium; action of an acid; increased amount of lime, forming with the pigments a nucleus; secretion of calcium, from the mucous membrane of the gall bladder. All stones, however, do not contain all these substances. Formation is chiefly due to the precipitation of some one substance, and this does not occur until the glycocholate or taurocholate of sodium decomposes, when the reaction is changed to acid.

In order that the concretions should be of any size, it is necessary for the bile to be retained in the gall bladder, or ducts, for some time. Sometimes erosions are found in the stones, caused, occasionally, by depositions; sometimes they divide, and these conditions make their disposal easier of accomplishment.

The influence of age on the formation of biliary calculi is difficult to understand, unless it be due to the habits and changes age brings. They are most common over twenty-five years. Females are more prone than males, three to two. Multiparity predisposes, as do diseased conditions anywhere; morbid changes in the liver and its passages, cancer, adhesions to various organs. Another cause is sedentary habits. Too long intervals between meals favors stagnation. It is doubtful if there exists a diathesis, predisposing to stone.

DISCUSSION.

DR. TAYLOR quoted Murphy, of Chicago, who says the gall bladder plays an insignificant part in the storage of bile. If this is not so, removal should be attended by disastrous results upon the system. In fact, it is not. It is always found filled with bile whose specific gravity varies from that of the liver. It subserves the purpose, says Murphy, of controlling the discharge—making it continuous—by keeping up tension, like the second bulb of an atomizer. A puncture of the gall bladder does not contract, and the bile continues to flow from it, demonstrating the absence or inefficiency of the muscular fibers.

DR. LANDON B. EDWARDS contended that if the bladder acts as a second bulb, it must do so continuously, causing it to empty itself, eventually. Then if a puncture allows the continuous escape of bile, where can it come from? The bladder may act as the first bulb, but, to his mind, the second bulb idea is erroneous.

MARK W. PEYSER, M. D.,
Secretary and Reporter.

---

HYPNOTISM IN SURGERY.—Fifty years ago Dr. L. A. Dugas, of Georgia, operated three times upon a woman in the hypnotic state for recurring carcinoma of the breast. In the third operation the axillary glands were also removed. The patient gave no indication whatever of sensibility (*Southern Medical and Surgical Journal*, March, May and September, 1845). Dr. Dugas believed that the phenomena of "mesmeric sleep" might be so utilized as to "render them available to all sufferers." A little earlier Dr. Crawford W. Long, the discoverer of anæsthesia, had investigated the subject and disused hypnotism because of its uncertainty of action. Hypnotism has thrice been revived and twice fallen into disuse during the last century.—*Medical Standard.*

